# Gastrointestinal Viral Load and Enteroendocrine Cell Number Are Associated with Altered Survival in HIV-1 Infected Individuals

**DOI:** 10.1371/journal.pone.0075967

**Published:** 2013-10-16

**Authors:** Guido van Marle, Keith A. Sharkey, M. John Gill, Deirdre L. Church

**Affiliations:** 1 Snyder Institute for Chronic Diseases, University of Calgary, Calgary, Alberta, Canada; 2 Hotchkiss Brain Institute, University of Calgary, Calgary, Alberta, Canada; 3 Department of Microbiology, Immunology & Infectious Diseases, University of Calgary, Calgary, Alberta, Canada; 4 Department of Physiology & Pharmacology, University of Calgary, Calgary, Alberta, Canada; 5 Department of Pathology & Laboratory Medicine, University of Calgary, Calgary, Alberta, Canada; 6 Division of Microbiology, Calgary Laboratory Services, Calgary, Alberta, Canada; Karolinska Institutet, Sweden

## Abstract

Human immunodeficiency virus type 1 (HIV-1) infects and destroys cells of the immune system leading to an overt immune deficiency known as HIV acquired immunodeficiency syndrome (HIV/AIDS). The gut associated lymphoid tissue is one of the major lymphoid tissues targeted by HIV-1, and is considered a reservoir for HIV-1 replication and of major importance in CD4+ T-cell depletion. In addition to immunodeficiency, HIV-1 infection also directly causes gastrointestinal (GI) dysfunction, also known as HIV enteropathy. This enteropathy can manifest itself as many pathological changes in the GI tract. The objective of this study was to determine the association of gut HIV-1 infection markers with long-term survival in a cohort of men who have sex with men (MSM) enrolled pre-HAART (Highly Active Antiretroviral Therapy). We examined survival over 15-years in a cohort of 42 HIV-infected cases: In addition to CD4+ T cell counts and HIV-1 plasma viral load, multiple gut compartment (duodenum and colon) biopsies were taken by endoscopy every 6 months during the initial 3-year period. HIV-1 was cultured from tissues and phenotyped and viral loads in the gut tissues were determined. Moreover, the tissues were subjected to an extensive assessment of enteroendocrine cell distribution and pathology. The collected data was used for survival analyses, which showed that patients with higher gut tissue viral load levels had a significantly worse survival prognosis. Moreover, lower numbers of serotonin (duodenum) and somatostatin (duodenum and colon) immunoreactive cell counts in the gut tissues of patients was associated with significant lower survival prognosis. Our study, suggested that HIV-1 pathogenesis and survival prognosis is associated with altered enteroendocrine cell numbers, which could point to a potential role for enteroendocrine function in HIV infection and pathogenesis.

## Introduction

Human immunodeficiency virus type 1 (HIV-1) infects and destroys cells of the immune system, which ultimately leads to CD4+ T-cell depletion and a profound immune deficiency known as Acquired Immunodeficiency Syndrome (HIV/AIDS) (reviewed in [1]). The gut associated lymphoid tissue (GALT), is one of the major lymphoid tissues targeted by HIV-1, and is considered the reservoir for HIV-1 replication and of major importance in CD4+ T-cell depletion [2], [3], [4], [5], [6]. HIV-1 infection is also associated with gastrointestinal (GI) dysfunction, which is directly caused by HIV-1 [7], and known as HIV enteropathy. Indeed, enteric opportunistic infections are common in HIV/AIDS [8], [9], [10], but symptoms of gut dysfunction frequently occur at earlier stages of HIV-1 infection in treatment naïve patients, underscoring the direct enteropathogenic nature of HIV-1 [7], [11], [12], [13], [14], [15]. The clinical indications of HIV-1 enteropathy include chronic diarrhea in the absence of other opportunistic enteric pathogens and progressive wasting or ‘slim disease’ [7], [16], [17], [18], [19].

Even though, HIV-related gut dysfunction and HIV associated mortality has been substantially reduced in the developed world since the introduction of highly active anti-retroviral therapy (HAART), many HIV-infected persons in resource-limited countries remain without treatment or receive suboptimal treatment. Evidence from both the SIV macaque infection model and more limited studies in HIV-1 infected humans show that the pool of activated memory CD4+ CCR5+ CD4 cells in the GALT effectors site (i.e. lamina propria) are substantially depleted during primary SIV and HIV infection [3], [20], [21], [22]. Further depletion of these immune cells occurs in GALT inductor sites (i.e. Peyer's patches and mucosal lymphoid follicles) as result of chronic HIV-1 infection and persistent mucosal antigenic activation of latently infected cells, such as CD4+ CCR5+ lymphocytes and macrophages [2], [23]. Persistent systemic immune activation in the GALT has been shown to lead to increased barrier permeability and bacterial translocation and the release of pro-inflammatory cytokines [21], [23]. Our group also found alterations in neuropeptide expression in the GI tract of HIV-1 infected individuals compared to uninfected controls, which may be associated with HIV enteropathy [24]. Inflammation has been shown to affect levels of serotonin and somatostatin producing cells in other disease contexts such as Crohn's disease or other gastrointestinal infections [25], [26], [27], [28], [29]. As chronic gastrointestinal inflammation is one of the hallmarks of HIV-1 associated disease, alterations in these enteroendocrine cells would be associated with HIV-1 enteropathy and disease progression. It would be logical to assume that patients with a direct HIV-1 enteropathy and increased HIV-1 activity in the gut have a worse prognosis. However, limited data are available, in particularly in minimally treated or treatment naïve HIV-infected patients that correlate viral activity and gut tissue markers of disease progression (i.e., CD4 and viral load levels, enteroendocrine function) and mortality. The goal of this study was to determine in a cohort of HIV-infected men who have sex with men (MSM), whether gut HIV-1 infection as measured by presence of virus, viral load, and viral phenotype, in two gut tissue compartments (duodenum and colon) were associated with altered survival outcome. We used current and historic data from patients enrolled in a comprehensive prospective study of gut dysfunction in the pre-HAART era at the Southern Alberta HIV Clinic (SAC), Calgary, Alberta. This cohort has been followed for more than 15 years, and we found that increased viral replication as well as altered enteroendocrine function was associated with reduced survival.

## Methods

### Study Population

The patient cohort used in this study has been described previously [12], [13], [24], [30], [31], [32], [33]. In brief, in 1991 a cohort of 42 HIV-infected men who have sex with men (MSM) and 8 HIV seronegative age and gender matched controls were enrolled at the Southern Alberta HIV Clinic (SAC) (Table 1). This prospective study focused on gut dysfunction during HIV infection. All patients gave written informed consent. All protocols and procedures have been reviewed and approved by the Conjoint Health Ethics Research Board (CHREB) at the University of Calgary and Alberta Health Services (Calgary). The patient samples were all collected before the introduction of highly active antiretroviral therapy (HAART) at the SAC in 1997. Patients were primarily on monotherapies or dual therapies consisting of nucleoside reverse transcriptase inhibitors (NRTIs), such as azidothymidine (AZT, zidovudine), 2′,3′-dideoxy-3′-thiacytidine (3TC, lamuvidine) and dideoxyinosine (ddI).

**Table 1:** Baseline comparison HIV-infected (HIV^+^) and control cases^1^.

	Controls (N = 8)	HIV^+^ (N = 42)	*p*-value
Age^2^	38.3 (29.3–58.0)	38.1 (31.9–42.9)	0.98
BMI^2^	26.3 (23.9–27.9)	25.1 (22.5–27.3)	0.39
CD4^+^ cells/µL^2^	696 (557–851)	245 (140–462)	0.0009
Plasma HIV VL (log_10_ copies/mL)^2^	N/A	3.93 (3.32–4.79)	-
Ideal Body Weight (kg)^2^	76 (72–78)	76 (72–79)	0.78
Skin-Fold (triceps) (cm)^2^	15.7 (7.4–28.4)	8.0 (6.3–10.0)	0.054
Lactulose-Mannitol test^2^	0.017 (0.012–0.027)	0.025 (0.020–0.035)	0.028
Lost weight^3^	4 (50)	21 (51)	0.95
Bowel pattern change^3^	0 (0)	19 (45)	0.016
Bowel movement/day ( = 1)^3^	1 (13)	3 (7)	0.36
Bowel movement/day ( = 2)^3^	5 (63)	17 (40)	-
Bowel movement/day (≥3)^3^	2 (25)	22 (52)	-
Diarrheal episode ( = 1)^3^	1 (13)	16 (76)	0.86
Diarrheal episode ( = 2)^3^	0 (0)	4 (19)	-
Diarrheal episode (≥3)^3^	0 (0)	1 (5)	-
Anti-diarrheal agent^3^	0 (0)	5 (13)	0.51
Distal sensory polyneuropathy^3^	0 (0)	9 (21)	0.15
HIV p24^+^ PBL Culture^3^	0 (0)	25 (60)	0.0020
HIV p24^+^ Duodenum Culture^3^	0 (0)	8 (19)	0.19
HIV p24^+^ Colon Culture^3^	0 (0)	17 (40)	0.15

1 Abrevations: BMI = body mass index; VL = viral load; N/A = not applicable; PBL = peripheral blood lymphocytes; p24^+^ = p24 antigen positive;

2 Median (interquartile range - IRQ);

3 N (%).

### Clinical Patient assessment and sample collection

Upon enrollment, we collected for each case clinical (GI symptoms, physical/neurological exam including weight, BMI and skin-fold caliper measurements) and laboratory test data (CBC and differential, absolute CD3-, CD4- and CD8-lymphocyte counts and plasma viral load measurement). Peripheral blood lymphocytes (PBLs) isolated from blood samples using standard procedures were stored in liquid nitrogen [30]. Gut permeability was assessed by the lactulose-mannitol absorption test as previously described [34]. Patients were assessed at every visit for any opportunistic or other infections. To detect enteric infections, stool cultures and ova & parasite tests were performed. Any known infections prior to endoscopy were treated with appropriate antimicrobial agents. Endoscopy and sample collection was performed at least a month after treatment, to reduce effects of the antimicrobial treatment. Following biopsy all patients were assessed for enteric infections, and patients were treated accordingly. These enteric infections at the time of biopsy were recorded for the pathology analysis. As described previously [24] upper and lower endoscopies were performed at every second visit (i.e. Visit 1, Visit 3, Visit 5 etc.) during the study period (i.e., approximately every 6–8 months). Biopsy forceps were used to harvest multiple (i.e., 4–6 pieces) gut tissue biopsies (i.e., average size = 10 mg ± 0.1). Biopsies from the duodenum (D) and distal colon (C) were placed in either viral transport media or fixative and directly transported to the laboratory for processing and/or storage at −70°C within 1 h after harvest.

### Virological Analyses

PBLs and gut tissue biopsies taken via an endoscopic procedure were stored in viral transport media and shipped within 1 hour of collection to the laboratory for coculturing. The biopsies were washed with culture media (RPM1 1640 medium containing fetal bovine serum and antibiotics) and mechanically disrupted before co-culturing with phytohemagglutenin (PHA-P) and IL-2 stimulated peripheral blood mononuclear cells (PBMCs) (1×10^6^ cells/well) from an HIV-1 seronegative donor in a 24 well plate. Media was harvested twice a week. Tissues were co-cultured for 14 days to a month, and media was harvested twice a week. Fresh PBMCs from a HIV-1 seronegative donor were added every week to keep the amount of PBMC at 1 to 2×10^6^ cells per well. HIV-1 p24 positive media (above 30 pg/ml) of the cocultured biopsies and PBL were collected and stored at −70°C until further analysis. This procedure has been described previously [30], [35]. HIV isolates were phenotyped as either non-syncytium-inducing or NSI (CCR5+) or syncytium-inducing or SI (CXCR4+) strains using a previously described procedure [31]. Briefly, supernatants from positive p24 (≥30 pg/ml) co-cultured PBLs and/or gut tissues (D/C) were added onto MT-4 lymphoblastoid cell cultures in RPM1 1640 medium containing fetal bovine serum, and cytopathological effects were recorded starting at day 3 for up to 14 days. HIV-1 reference strains as positive and negative control cultures were included in each assay plate. The HIV reference strains used were HIV IIIB (HTLV IIIB) and HIV RF, and were obtained from the National Institutes of Health AIDS reagent program (www.aidsreagent.org).

Levels of HIV-1 RNA in plasma were quantified retrospectively on stored samples by the Amplicor HIV-1 Monitor v1.5 assay (Roche Molecular Diagnostic Systems, Branchburg, NJ) using polymerase chain reaction according to the manufacturer's instructions. Levels of HIV-1 gut tissue RNA were quantified by the NASBA (Nucleic Acid Sequence Based Amplification) HIV-1 RNA QT assay (formerly Organon Teknika, Durham, N.C.), according to the tissue protocol supplied by the manufacturer. Gut tissue HIV-1 RNA viral load measurements were recorded as log_10_ copies per mg of tissue.

### Histological Analyses

Gut tissues were blotted on sterile filter paper to remove any mucus or secretions immediately after collection, and directly fixed in cold Zamboni's fixative as described previously [24]. The tissues were fixed at 4°C for 12–18 hours. After fixation, tissues were washed, 1 time in PBS (phosphate buffered saline, pH 7.4), 3 times for 10 minutes in dimethyl sulfoxide (DMSO), 3 times for 10 minutes in PBS (pH 7.4), and finally left in PBS (pH 7.4) with 20% sucrose. The cryopreserved tissues where subsequently imbedded in Tissue Tk* O.C.T. compound and sectioned on a cryostat (Leica, Germany). Sections of duodenal and colonic tissues of each patient were mounted side by side on a slide, to ensure consistent staining between the tissues. Sections were processed for indirect immunofluorescence detection of serotonin (5-HT, 5-hydroxy tryptamine), and somatostatin immunoreactive cells using the antibodies and procedures described by Sharkey *et al.* [24]. Fluorescent secondary antibodies were obtained from DAKO Inc. (Burlington, ON, Canada). For each visit and each patient slides were coded and scored by two observers blinded for the identity of the slides. They independently scored the slides qualitatively for staining intensity, as well as enumerated all serotonin and somatostatin immunoreactive cells per slide, by counting multiple views per section per slide (magnification 40×). All cell numbers were expressed as cells/mm^2^. The scores assigned by the two observers corresponded in almost all cases. In case of disagreement between observers, slides where reassessed by the (blinded) observers. Tissue sections for the duodenum were also subdivided in villi, glandular mucosa, and muscularis mucosa, and for the colon in mucosa and muscularis mucosa. Similarly, pathological analyses were performed and sections were assessed for parameters such as villus atrophy and inflammatory cell invasion using previously described protocols [24].

### Statistical Analysis

All data was analyzed using statistical analysis software (SPSS for Windows version 14.0 and 20) and STATA 9.0 (Stata Corp., College Station, TX, USA). Prior to analysis all continuous variables were assessed qualitatively using histograms. Non-normally distributed variables were described using medians with interquartile ranges (IQR) and compared using the Mann-Whitney U test. For continuous variables multiple group-by-group comparisons were performed using ANOVAs with appropriate post-hoc test. Survival data was presented as Kaplan-Meier plots. The survival functions were compared using the log-rank test and cox-regression. Some analyses involved developing a population-averaged panel-data model using a generalized estimating equation (GEE) method (linear model) [36] to assess individual error estimation of unequal variance because each individual provides a variable number of data points based on their outcome and length in the study.

## Results

### Patient Population Characteristics

**Figure 1:**
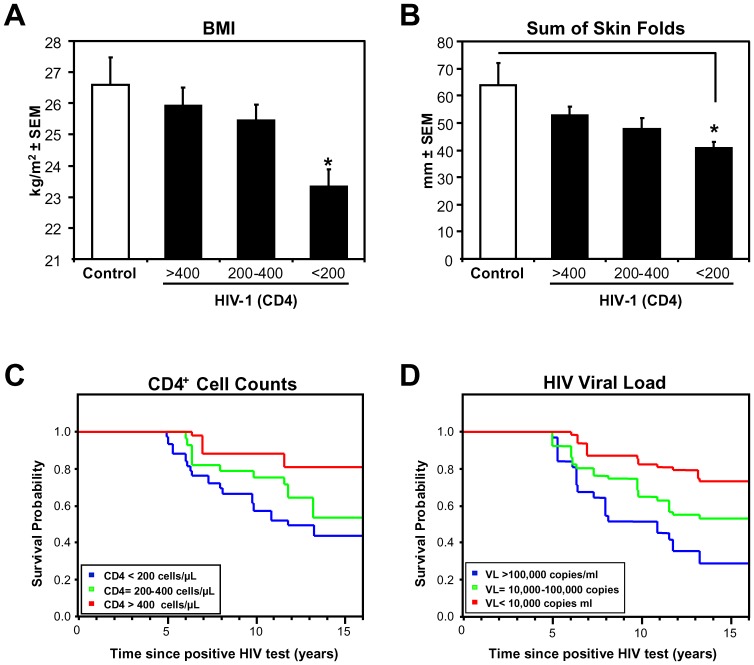
Patient Population Characteristics. Body mass index (BMI) (**A**) and sum of skin folds (**B**) of HIV infected individuals grouped by CD4+ cell count (CD4>400 cells/µL (n = 10), 400 to 200 cells/µL (n = 7), <200 cells/µL (n = 19)) compared to uninfected controls (n = 8), and Kaplan-Meier plots of our cohort of HIV infected individuals grouped by CD4+ cell counts (CD4>400 cells/µL (n = 13), 400 to 200 cells/µL (n = 10), <200 cells/µL (n = 19)) (**C**) and viral load (VL) (VL>100,000 copies/ml (n = 14), VL = 10,000–100,000 copies/ml (n = 10), <10,000 (n = 18)) (**D**) BMI and sum of skin folds was reduced in HIV infected individuals with a CD4+ T cell count <200 cell/µL, and survival probability decreased with decreasing CD4+ cell counts and increasing viral load. These results show that the patients in our cohort progressed towards disease normally. (*p*<0.05, Dunnet's post hoc test).

Table 1 shows baseline characteristics of the 42 HIV-infected cases and 8 controls. Upon enrollment cases had been HIV-1 seropositive for a median of 4 years (interquartile range; IQR; 1–5). There was no difference in the time between study enrollment or the ages of cases or controls, but cases had a median plasma viral load of 3.93(IQR; 3.32–4.79) log_10_copies/mL and significantly lower absolute CD4 counts. Although there was not a significant difference in the ideal body weight or BMI or skin-fold caliper measurements (triceps) between controls and all HIV-infected individuals combined, we observed significant decreases in BMI and sum of skin folds in patients with low CD4 counts (<200 cell/µl) (Fig. 1A and B, p<0.05) indicative of GI dysfunction typically observed in advanced HIV-1 infection [7], [37]. Cases were also more likely to report bowel dysfunction including bowel pattern changes, multiple bowel movements/day, altered lactulose manulose (lacman) absorption test for intestinal permeability, taking an anti-diarrheal agent or have distal sensory polyneuropathy (Table 1). HIV was cultured at baseline from peripheral blood lymphocytes and/or gut tissue from most cases. At baseline, 10 patients were on AZT monotherapy, 20 were on DDI monotherapy, three were on AZT/DDI combination therapy and one patient was treated with 3TC, AZT and DDI combination therapy. Patients were followed prospectively for a median of 5.4 yrs. (IQR; 2.0–12.2 yrs.): 19 (45%) died, 13 (31%) survived until the end of the study and 10 (24%) were lost to follow-up.

The median patient baseline CD4 cell count/µl was 245 (IQR; 140–462), the CD8 count/µl was 849 (IQR; 561–1188) and the CD48 count/µl was 300 (IQR; 150–250). There was a significant CD4 count declined over the study period (p<0.001). As expected lower survival probability was significantly associated with lower CD4 count (Figure 1C, *p*<0.001). The median baseline plasma HIV viral load for cases is 3.93 log_10_ copies/ml (IQR; 3.32–4.79 log_10_ copies/ml). A significantly higher median plasma VL was found in those patients with a CD4 cell count <200 cells/µl (VL = 4.30 log_10_ copies/ml; IQR; 3.78–4.99 log_10_ copies/ml) compared to those cases with higher absolute CD4 counts (VL = 3.48 log_10_ copies/ml; IQR; 2.79–4.51 log_10_ copies/ml; *p*<0.05). There was no significant rate of change of VL over time between cases that died vs. those that survived (p = 0.92), but lower survival probability was significantly associated with higher plasma VL at the start of the study (Figure 1D, *p*<0.001). We could not find an association between the use of anti-retroviral therapy (ART) and change in VL, consistent with the suboptimal preHAART treatments patients received at the time of these studies. Taken together these observations showed our study population had the standard progression towards disease, and was not enriched for either rapid or long term slow progressors.

### Increased viral activity in the gut is associated with lower survival

Twenty-nine patients had positive HIV-1 cultures in one or more compartments; 25 blood samples (60%) tested positive in peripheral blood lymphocytes, 8 (19%) in duodenum, and 17 (40%) in colon biopsies (Table 1). The median plasma viral load was significantly higher among those patients for whom virus could be cultured from tissue biopsies (VL = 4.51 IQR; 3.69–4.92 vs. 2.79 IQR; 2.70–3.53; *p*<0.0001). Viral phenotypes were either CCR5 in 20 (48%), CXCR4 in 5, or not measurable in 17 (40%) out of 42 cases in blood isolates. For the duodenum, 8 were CCR5 (20%), 1 was CXCR4 (2%), and 32 (78%) out of 42 were unmeasurable, while for the colon 15 (38%) were CCR5, 3 (8%) were CXCR4, and 22 (55%) out of 42 cases were unmeasurable. Median viral load was significantly higher in the colon (6.43 log_10_ copies/mg/tissue; IQR; 3.70–6.60 log_10_ copies/mg tissue) than in the duodenum (3.70 log_10_ copies/mg/tissue; IQR; 3.58–4.96 log_10_ copies/mg tissue; *p*<0.001).

**Figure 2:**
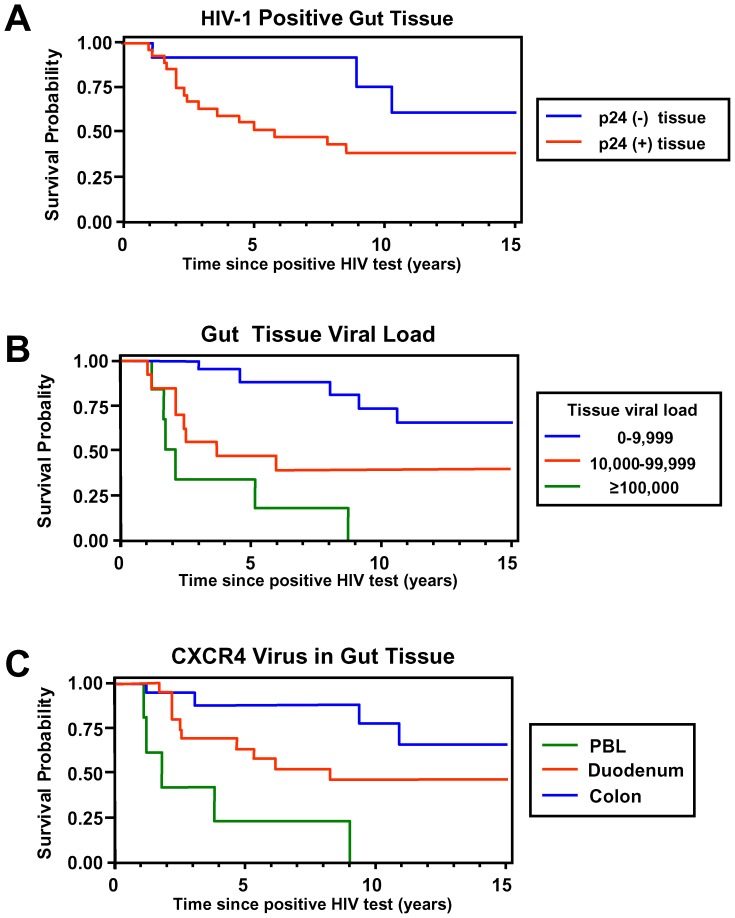
Kaplan-Meier plots of survival probability based on HIV-1 viral parameters. Kaplan-Meier plots of survival probability based on detection of HIV-1 p24 in any gut tissue (duodenum and/or colon) (p24(+) n = 25, p24(−) n = 17)(**A**) gut tissue viral load (**B**) and presence of CXCR4 using HIV-1 strains in peripheral blood lymphocytes (PBL), duodenum and colon (**C**). Detection of HIV-1 p24 and increased viral load in any gut tissue was associated with reduced survival. Although a low number of viral isolates were obtained from the different tissues (PBL 25, duodenum 8, colon 17), analysis of the presence of different viral strains in the different gut tissues affected survival differently, suggesting lower survival estimates associated with CXCR4 strains recovered from the duodenum compared to the colon. These data suggest and are consistent with previous observations that increased viral activity as well as viral diversity in the gut and different gut tissues is associated with HIV disease progression.

Almost one-half of the cases died during the 5-year study period. A number of plasma and gut tissue compartment features identified at baseline were associated with different survival functions. Evidence of an active gut HIV-1 infection (i.e., any positive HIV gut tissue culture) was associated with a worse prognosis than those patients in whom HIV was not isolated from any of the gut biopsies (Figure 2A).

Higher gut tissue viral load levels were associated with a significantly worse prognosis and cases with the highest baseline gut tissue VL; cases with >5.0 log_10_ copies/mg tissue had lower survival probability than those with lower gut tissue viral replication (*p*<0.0001) (Figure 2B).We also assessed survival of patients for whom their HIV isolates had a CXCR4 phenotype at one or more tissue sites is shown in Figure 2C. The numbers of isolates obtained was small and analysis of the recovery of CXCR4 HIV-1 strains from in particular peripheral blood lymphocytes suggested an association with a lower survival probability (*p*<0.05) consistent with previous observations [38], [39], [40], [41], while recovery of these strains from the duodenum was associated with lower survival probability compared to the colon (p<0.05).

### Diminished serotonin and somatostatin expressing enteroendocrine cells in the gut of HIV-1 infected individuals

**Figure 3:**
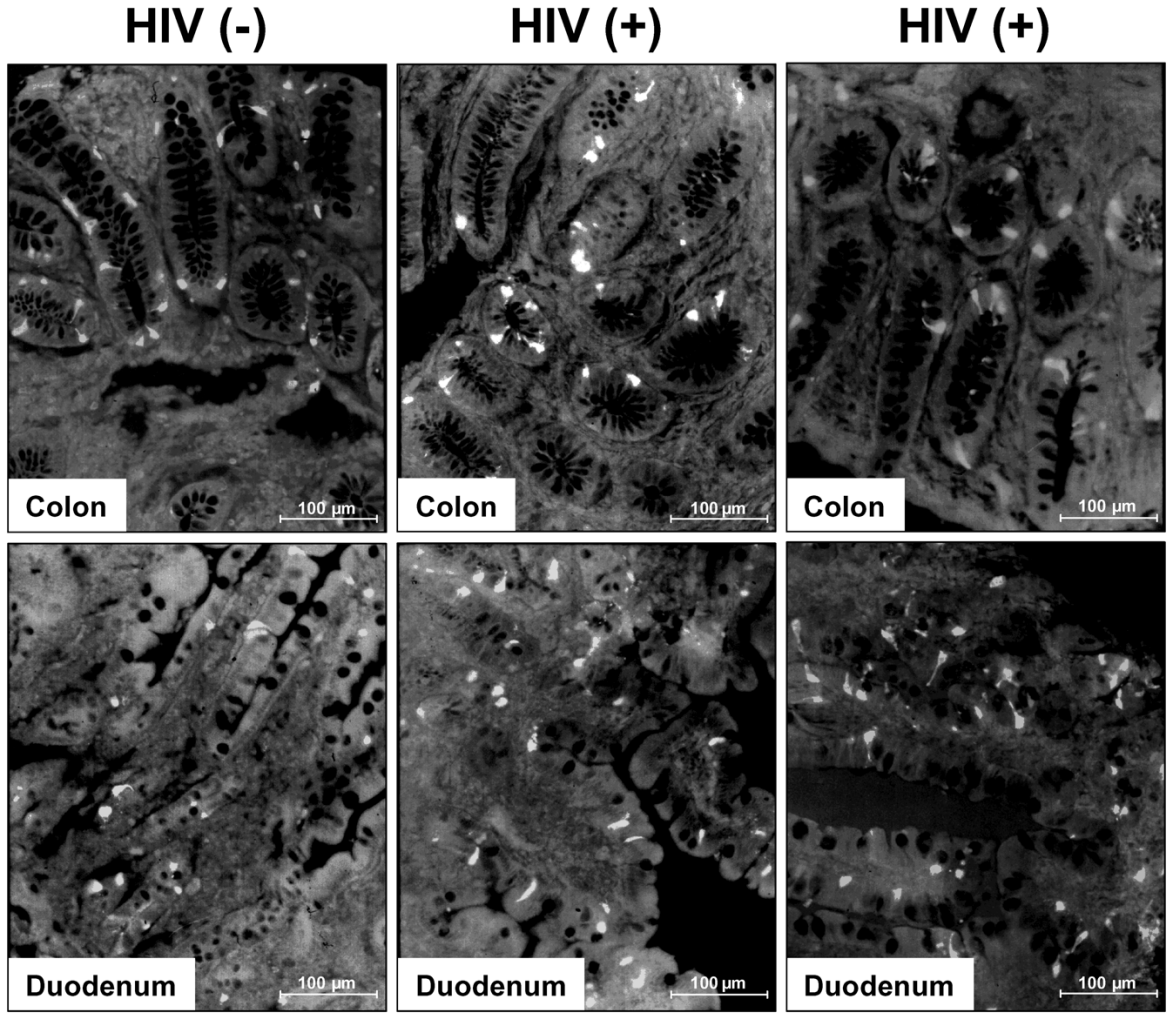
Immunofluorescence analyses of enteroendocrine cells in the duodenum and colon in HIV-1 infection. Representative immunofluorescence analyses of serotonin immunoreactive cells in the duodenum and colon of 2 HIV infected individuals (HIV(+)) and 1 uninfected control patient (HIV (−)). Number of serotonin immunoreactive cells differed amongst different HIV infected individuals and tissues as well as compared to uninfected controls.

**Figure 4:**
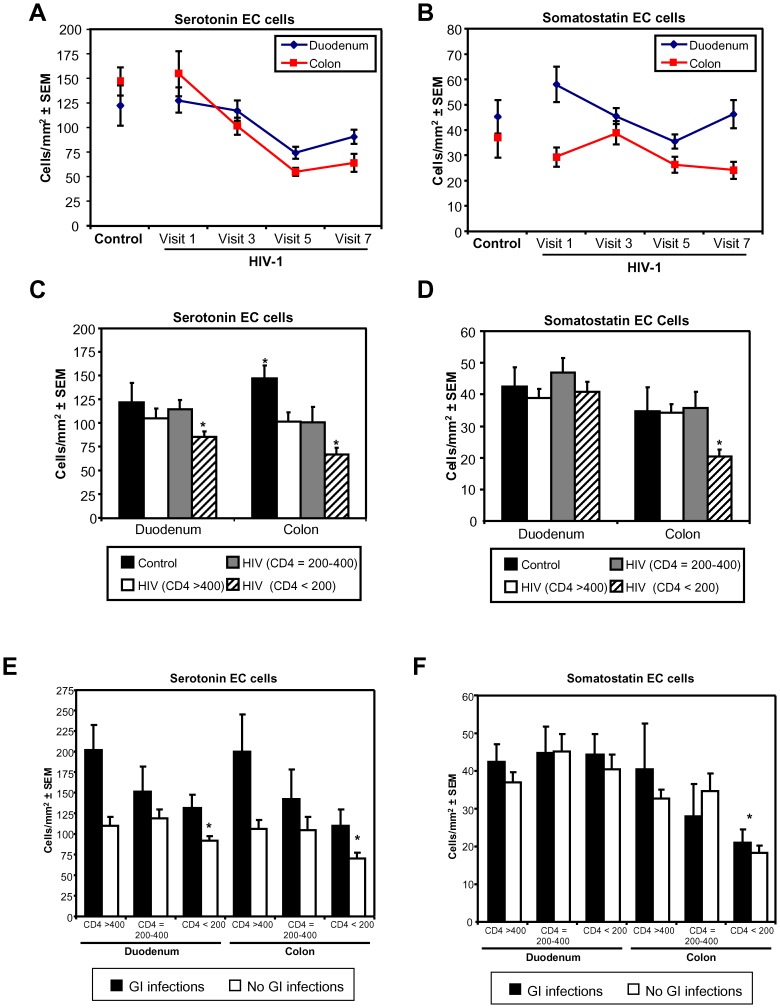
Number of enteroendocrine cells in the duodenum and colon of HIV-1 infected and control patients. Serotonin (**A**) and somatotstatin (**B**) immunoreactive cell numbers changed over the course of infection in the duodenum and colon of HIV-1 infected individuals. (**C**) In both duodenum and colon serotonin cell numbers was significantly reduced in HIV-1 infected patients with CD4+ T-cell counts below 200 cells/µl, while in the colon the number of cells was reduced in all HIV-1 infected individuals compared to uninfected controls. (CD4>400 cells/µL (n = 10), 400 to 200 cells/µL (n = 7), <200 cells/µL (n = 17), controls (n = 8)) (**D**) Somatostatin cell numbers were significantly decreased in the colon of HIV-1 infected individuals with CD4+ T-cell counts below 200 cells/µL. (CD4>400 cells/µL (n = 10), 400 to 200 cells/µL (n = 7), <200 cells/µL (n = 18), controls (n = 8) (**E**) Serotonin cell numbers were higher in biopsy samples (CD4>400 cells/µL (n = 12), 400 to 200 cells/µL (n = 8), <200 cells/µL (n = 24) of HIV-1 infected patients with GI infections at the time of biopsy compared to biopsies of patients without GI infections in both duodenum and colon. However, for both situations enteroendocrine cell numbers decreased with lower CD4+ T-cell levels. (**F**) GI infections did not appear to have an affect somatostatin enteroendocrine cell numbers, but somatostatin numbers were decreased in both groups of HIV-1 infected patients with a CD4+ T-cell counts lower than 200 cells/µL (* *p*<0.05 Dunnet's post hoc test).

To determine if high viral load in the gut tissues, was associated with any GI abnormalities, we subjected the tissues to an extensive immune and histopathological analysis. Contrary to earlier pilot studies, we did not observe an overt inflammatory cell invasion or villus atrophy (duodenum), associated with the increased gut tissue viral load. Serotonin (5-HT) and somatostatin are enteroendocrine molecules important for GI motility and barrier function, and a careful balance needs to be maintained for normal GI function (reviewed in [25], [27], [28], [42], [43]). Enumeration of the serotonin (Figure 3) and somatostatin enteroendocrine cells in the duodenum and colon, revealed a decrease in serotonin cells over time in both tissues (Figure 4A), while a similar though less consistent trend was observed for the somatostatin cells (Figure 4B).

Excluding cases with known GI infections and grouping these cases by CD4+ T-cell count (CD4+ cell count >400 cells/µL, between 400 and 200 cells/µL, and <200 cells/µL) we did observe lower numbers of serotonin cells in the duodenum and colon of patients with a CD4+ T-cell count of <200 cells/µL (Figure 4C, *p*<0.05), while a reduction of somatostatin cells was only observed in the colon in this group of patients (Figure 4D, *p*<0.05). Moreover, a reduction in serotonin cells was observed in the colon for all HIV-1 infected individuals compared to healthy controls (Figure 4C, *p*<0.05). As many GI pathogens can upregulate enteroendocrine peptides such as serotonin, we also compared the number of serotonin and somatostatin immunoreactive cells, in the HIV-1 infected individuals with and without known GI superinfections. Although we did see a higher number of serotonin cells in samples of both duodenum and colon in HIV-1 infected patients with GI infections compared to the samples with no GI infections, the number of serotonin cells was progressively lower in patients with a lower CD4+ cell count (Figure 4E, *p*<0.05). The GI infections did not significantly alter the number of somatostatin immunoreactive cells (Figure 4F). Similar to the HIV-1 patients without GI infections, the number of somatostatin immunoreactive cells were significantly reduced in the colon with a CD4+ cell count below 200 cells/µL (Figure 4E and F, *p*<0.05).

We assessed if these lower enteroendocrine cell counts were correlated with any other clinical/pathology observations. We did not observe any direct associations with overt villus atrophy, inflammatory cell infiltration, diarrhea or altered BMI. Although a reduction in the sum of skinfolds was associated with lower serotonin immunoreactive cell counts in the colon, no direct link to high or low plasma or gut viral load was found (data not shown).

### Altered serotonin and somatostatin enteroendocrine cell numbers are associated with lower survival

**Figure 5:**
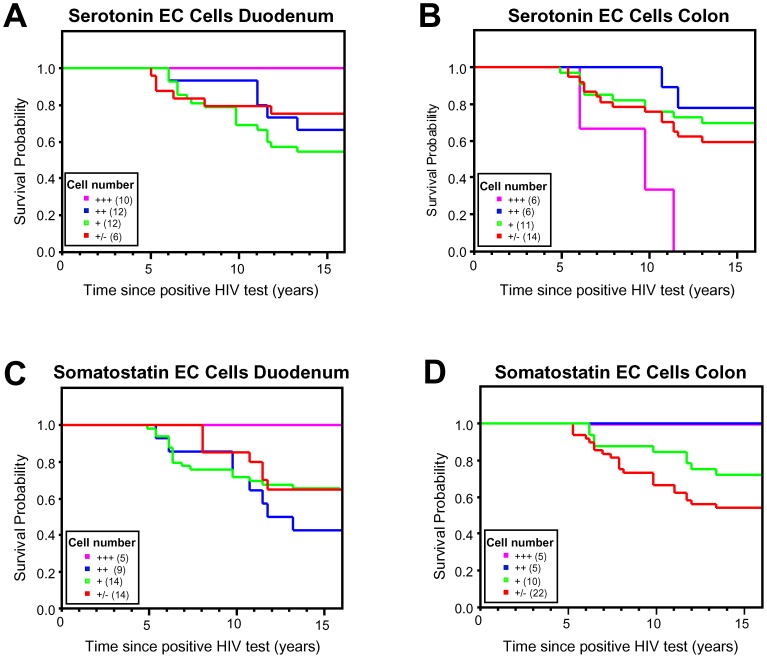
Kaplan-Meier survival plots based enteroendocrine cell numbers in duodenum and colon of HIV-1 infected patients. (**A**) Reduced serotonin cell numbers (++, +, +/−) in the duodenum was associated with lower survival probability compared to patients with high serotonin cell numbers (+++). (**B**) In contrast, high serotonin cell numbers (+++) in the colon were associated with a significant reduced survival probability compared to each of the other groups (*p*<0.05). (**C**) Reduced somatostatin cell numbers (++, +, +/−) in the duodenum was associated with reduced survival probability compared to patients with high cell counts (+++) (*p*<0.05). (**D**) Lower number of somatostatin cell numbers (+ and +/− respectively) was associated with a significant lower survival probability compared to the other groups of HIV-1 infected patients (+++ and ++)(*p*<0.05). (n numbers are indicated in graph inserts: serotonin cells: +++>180 cells/mm^2^, ++ = between 180 and 120 cells/mm^2^, + = between 120 and 60 cells/mm^2^ and +/−<60 cells/mm^2^, somatostatin +++>90 cells/mm^2^, ++ = between 90 and 60 cells/mm^2^, + = 60 and 30 cells/mm^2^ and +/−<30 cells/mm^2^).

Gastrointestinal alterations have been closely associated with various aspects of HIV-1 disease [7]. As we found altered enteroendocrine cell numbers in the GI tract of HIV-1 infected individuals was closely linked to CD4+ T-cell counts , we examined if lower enteroendocrine cell numbers in the colon and duodenum, were in any way associated with altered survival probability. Categorizing patients based on cell numbers (serotonin cells: +++>180 cells/mm^2^, ++ = between 180 and 120 cells/mm^2^, + = between 120 and 60 cells/mm^2^ and +/−<60 cells/mm^2^, somatostatin +++>90 cells/mm^2^, ++ = between 90 and 60 cells/mm^2^, + = 60 and 30 cells/mm^2^ and +/−<30 cells/mm^2^), revealed an association with a reduced survival prognosis of patients with lower numbers of serotonin (duodenum) and somatostatin (duodenum and colon) immunoreactive cell counts in their gut tissues compared to the group with high (+++) enteroendocrine cell counts (Figure 5A, C and D, *p*<0.05). A similar trend was observed in the colon for somatostatin cells (Figure 5D), where patients with a lower number of somatostatin cells appeared to have a lower survival probability. However, we also observed that patients with high (+++) serotonin cell counts in the colon (which were higher than controls) had an significant lower survival probability (*p*<0.05), suggesting excessive activation of enteroendocrine function might also be an important factor affecting HIV-1 pathogenesis.

## Discussion

The GI tract is a major target and reservoir for HIV-1 replication, but many assumptions regarding HIV-1 infection of the gut have been based on the extrapolation of observations observed in the SIV model and acute HIV infection [2], [3], [4], [5], [6]. It has been well established that the persistence of a high plasma viral load after primary HIV-1 infection is associated with a worse prognosis [44]. However, very few prior studies have been able to directly correlate the natural history of direct HIV-1 enteropathy and viral replication in the GI tract, in HAART naïve patients with disease progression. Our study demonstrates that signs of HIV-1 enteropathy and high levels of HIV activity in the GI tract is associated with disease progression and worse survival prognosis.

In our patients HIV-1 viral load levels in both the duodenum and colon were significantly higher (i.e., 2–3 logs) than those found in the plasma, a result that confirms and extends the findings of previously published studies [5], [32], [45], [46], [47]. Gut tissue viral load levels were significantly higher in both the upper and lower bowel tissues compared to those in plasma. ART monotherapy had no effect on duodenum VL levels overtime and there was the indication of a trend for ART to decrease the colonic viral load (*p* = 0.07), but this effect was not sustained over time (data not shown). A trend towards correlation between the HIV VL levels in plasma and colonic tissue overtime was observed, as well a trend towards increased colonic VL and decreased CD4 counts overtime but these were not statically significant (data not shown). No association was found between plasma VL or the peripheral blood CD4 count and gut tissue VL overtime, again underscoring previous observations that the different tissues behave independently in their replication dynamics and potential reservoirs for viral replication [32], [46], [48]. A potential limitation of our approach was the use of different HIV-1 viral load assays to quantify virus in plasma compared to both of the tissue sites. However, in previous studies we found significant inhibition using PCR approaches to quantify HIV-1 in gut tissues, while NASBA has been shown to amplify even partially degraded nucleic acid from stored tissues [49]. In addition, studies using paired plasma samples showed excellent correlation between these two assays yielding a mean difference in HIV RNA levels of 0.28 and 0.54 log10 copies/ml by the HIV-1 Monitor test and the NASBA assay respectively [50], indicating that our approaches were appropriate.

Our study is one of the first to show in the human setting that high gut tissue viral load is associated with disease progression and increased mortality. Previously, it was an assumption that HIV viral colonic load affected survival, based mainly on the observations in SIV [19]. Since the likely route of primary HIV-1 infection in this MSM cohort was anal intercourse and colorectal virus inoculation, higher initial and ongoing viral replication at this site may be explained in this way. However, our observations can also be extended to vaginal HIV-1 transmission, where the cells in the GALT are also an important target for HIV-1 early in infection [3], [6]. Human colorectal cells have been shown to be some of the most permissive to HIV-1 infection in vitro, and in vivo may promote higher levels of HIV-1 infection of mucosal epithelial cells at this site [51]. Alternatively, the colonic immune response to HIV-1 infection may be different than that in the upper gut regions and/or there may be unique enteric adaptation of HIV-1 quasispecies that promote tissue tropism and replication at this site, as indicated by our recent studies on HIV-1 evolution in the gut compartments [32], [33].

The number of viruses recovered was very low, and our analysis of co-receptor use of virus from the GI tissues may suggest that viral diversity in these compartments affect disease outcomes differently. Primary HIV-1 infection targets CCR5+ immune cells, as the disease progresses, alternate co-receptors such as CXCR4 may become predominant due to the loss of the accessible CCR5+ in the cellular pool [52], [53], [54]. This is consistent with the notion that evolution of the viral infection to produce HIV-1 strains that can readily utilize CXCR4+ cells, either in the plasma or gut tissue compartments, herald rapid disease progression to death [54]. Previous work has shown that a switch in predominance of a particular viral phenotype based on CXCR4 co-receptor use is more complex [40] and we may not observe this in the current context of HAART. Indeed, it has been shown that other viral genotypic and phenotypic switches occur in one or more compartments over the course of infection, underscoring that dominance of different viral populations are linked to different states of illness [55], [56]. Moreover, in the context of co-receptor use, even subtle changes within the interaction between the viral envelope protein and the CCR5 receptor, without switching to CXCR4 use, can result in activation of a differential pathogenic signaling cascade, as has been clearly demonstrated in the context of HIV-1 associated neurological diseases [57]. Due to suboptimal treatment our patients all progressed toward disease in our study, and the biological reason for evolution to a CXCR4+ phenotype in our patient population may be the results of a survival advantage to the virus as the CCR5+ cell pool is depleted at a given site. There may also be trafficking of a particular viral phenotype from the blood to the different tissue compartments and vice versa over time [5]. However, it needs to be stressed that our data set was small, and we need to be cautious with extrapolating our observations to the HIV-1 infection in general

Our study also suggests that HIV-1 pathogenesis and survival prognosis is not solely associated with increased viral replication in the gut tissue compartment. We previously reported altered expression of various neuropeptides in the GI tract compared to uninfected controls in this cohort of HIV-1 infected individuals [24]. However, at that time we did not have any long-term survival data available to assess the impact of these changes. Our current observations suggest that alterations in the number of somatostatin and serotonin enteroendocrine cells in the duodenum and colon are associated with altered survival probability. Increased intestinal permeability (“leaky bowel”) in HIV-1 infection has been considered a hall mark and key player in HIV/AIDS disease progression. It has been postulated that bacterial products leaking/translocating from the lumen across the GI epithelial cell layer, results in local and systemic immune activation, which is a significant contributing factor to CD4+ immune cell depletion [21], [23]. In our patient cohort we did observe a more leaky bowel, albeit rather modest, as assessed using the lactulose mannitol absorption test for barrier dysfunction (Table 1) [34]. However, this was not associated with altered survival probability. Somatostatin and serotonin are important for GI motility and barrier function, and deregulation has been associated with diarrhea, constipation and barrier disruption [7], [27], [28]. Therefore, our observations may still be consistent with the hypothesis that bacterial translocation contributes to HIV/AIDS disease progression, but that it may not occur because of an overt leaky bowel, but rather be the consequence of a more subtle barrier dysfunction caused by enteroendocrine dysregulation. The subtlety of the GI pathology may also explain why we did not observe a direct correlation with significant motility issues (diarrhea or constipation) in our patient cohort. The age of the samples (>15 years ) did not allow us to measure bacterial products in blood, but it will be of interest to determine if for instance increased LPS levels as well as inflammatory markers (such as sCD14) in the blood are linked to low enteroendocrine cell numbers in newly or chronic infected individuals. The latter analyses would also give further insight in the magnitude of barrier dysfunction.

Our observations suggested that lower numbers of somatostatin cells in the duodenum and colon and serotonin cells in the duodenum were associated with lower survival probability, but we also observed that very high numbers of serotonin immunoreactive cells in the colon was associated with worse survival probability compared to patients with lower serotonin enteroendocrine cell numbers. It needs to be noted that this group only consisted of 6 patients compared to some of the other groups that contained 10 or more. Increase in serotonin producing cells has been reported in GI inflammation, and other pathologies of the GI tract associated with diarrhea particularly in context of other GI infections and chemotherapy (reviewed in [25], [28], [43]). We cannot exclude undiagnosed GI infections, but we believe that in the case of these patients this over activation could be the direct result of HIV-1 infection, indicating that multiple factors are at play.

Our data does not allow us to demonstrate causality between altered enteroendocrine cell numbers and survival. A multivariate analysis did not reveal any independent association of blood CD4^+^cell counts, serotonin and somatostatin cell numbers and survival. The observed effects could therefore be a bystander effect of systemic or tissue specific CD4+ T-cell depletion, which need further investigation. We were not able to measure CD4+ T- cell numbers in the gut tissue samples available in order to establish associations between tissue CD4+ cell levels and enteroendocrine cell numbers. CD4+ T cells have been shown to affect enteroendocrine cells. In SCID mice (serotonin/5-HT) enteroendocrine cell numbers increased significantly, when they were reconstituted with CD4+ T cells from immune competent mice infected with the nematode *Trichuris muris*, which appeared to be linked to Th2 cytokines [58]. The latter is consistent with another infection study using this parasite demonstrating a link between Th1 and Th2 cytokine environments and enteroendocrine cells [59]. In rhesus macaques CD4+ T cells and (serotonin/5-HT) enteroendocrine cells where found in very close proximity in the gut mucosa and demonstrated cell-to-cell contact [60]. Thus both the cytokine milieu and direct cell to cell interactions may play a role. In addition to CD4+ T- cell depletion, HIV infection results in a chronic inflammatory state, which includes the GI tract. Inflammation is important in the pathology of GI diseases, such as celiac disease, Crohn's disease or ulcerative colitis. In these diseases significant alterations in enteroendocrine molecules such as somatostatin and serotonin are observed [25], [26], [27], [28], [29]. Some of our patients were treated with mono or sometimes duo NRTI therapies, that potentially affect the GI mucosa, but our analysis did not reveal an association between therapy and enteroendocrine cell numbers. Therefore, we believe that the chronic inflammation and immune activation in HIV-1 infection is the likely one of the drivers of the changes we observed in our patients. Further investigation into how direct interactions with CD4+ cells and inflammation affect enteroendocrine cell function in HIV infection is warranted, in order to assess a pathogenic role of these cells in HIV-1 pathogenesis.

A limitation of our study is the small size of our cohort and the fact that patient status would have changed over time, most notably due to altering treatments and the later introduction of HAART. However, we chose the patient's clinical status and gut HIV infection status at baseline to evaluate the effect on long-term survival outcome, to mitigate the effect of HAART later in the disease course. The collection of the data for our study was completed in 1993, and the median time of HIV seropositivity for our patient cohort was 4 years at the time of enrolment. HAART was introduced only in 1997 at the Southern Alberta Clinic, and the majority of the patients passed away before that time. We therefore feel that our analyses were not significantly affected by the number of patients that survived longer due to the introduction of HAART in 1997.

HIV-1 and SIV enteropathy can be defined as overt pathological changes, such diarrhea, barrier dysfunction (leaky bowel), wasting, villus atropy [7]. However, even though our cohort was relatively small our study in these HIV-1 infected individuals on ART shows that HIV enteropathy can also manifest itself as subtle changes in neuroendocrine function as reflected in altered enteroendocrine cell numbers in the absence of overt GI pathology, that were associated with significantly lower survival probability. Moreover, none of our patients in the cohort were acutely infected, which may explain why we observed a more subtle pathology, and did not observe the overt GI pathology (enteropathy) as seen in acute infection [3], [6].

A recent review of various studies on the effects of HAART on the immune reconstitution (CD4+ cells) in the GALT by Costiniuk et al. [61], clearly suggest only partial restoration of the immune system in the GI tract by HAART, and suggests HAART alone is not sufficient. Combining HAART with immunomodulatory therapies could help immune restoration. Our study underscores the role enteric HIV-1 infection to disease progression and survival. However, it also suggests that we may need to focus on factors beyond the direct viral and immunopathogenic aspects of disease in this GI compartment. Regular monitoring of immune status and viral loads of readily accessible gut tissue compartments, such as the colon, extended with an analysis of enteroendocrine markers could be a valuable predictor of disease progression in naïve and HAART treated patients. In addition, when developing and assessing the success of new treatment interventions, the enteroendocrine status may need to be taken into consideration.

## References

[1] Lane HC (2010) Pathogenesis of HIV infection: total CD4+ T-cell pool, immune activation, and inflammation. Top HIV Med 18: 2–6. 20305309

[2] Lackner AA, Mohan M, Veazey RS (2009) The gastrointestinal tract and AIDS pathogenesis. Gastroenterology 136: 1965–1978. 19462506 10.1053/j.gastro.2008.12.071 PMC3755960

[3] Li Q, Duan L, Estes JD, Ma ZM, Rourke T, et al (2005) Peak SIV replication in resting memory CD4+ T cells depletes gut lamina propria CD4+ T cells. Nature 434: 1148–1152. 15793562 10.1038/nature03513

[4] Guadalupe M, Sankaran S, George MD, Reay E, Verhoeven D, et al (2006) Viral Suppression and Immune Restoration in the Gastrointestinal Mucosa of Human Immunodeficiency Virus Type 1-Infected Patients Initiating Therapy during Primary or Chronic Infection. J Virol 80: 8236–8247. 16873279 10.1128/JVI.00120-06 PMC1563811

[5] Sankaran S, Guadalupe M, Reay E, George MD, Flamm J, et al (2005) Gut mucosal T cell responses and gene expression correlate with protection against disease in long-term HIV-1-infected nonprogressors. Proc Natl Acad Sci U S A 102: 9860–9865. 15980151 10.1073/pnas.0503463102 PMC1159164

[6] Mattapallil JJ, Douek DC, Hill B, Nishimura Y, Martin M, et al (2005) Massive infection and loss of memory CD4+ T cells in multiple tissues during acute SIV infection. Nature 434: 1093–1097. 15793563 10.1038/nature03501

[7] Kotler DP (2005) HIV infection and the gastrointestinal tract. Aids 19: 107–117. 15668535 10.1097/00002030-200501280-00002

[8] Janoff EN, Smith PD (1988) Perspectives on gastrointestinal infections in AIDS. Gastroenterol Clin North Am 17: 451–463. 3049355

[9] Batman PA, Kotler DP, Kapembwa MS, Booth D, Potten CS, et al (2007) HIV enteropathy: crypt stem and transit cell hyperproliferation induces villous atrophy in HIV/Microsporidia-infected jejunal mucosa. Aids 21: 433–439. 17301561 10.1097/QAD.0b013e3280142ee8

[10] Kotler DP (1995) Gastrointestinal manifestations of human immunodeficiency virus infection. Adv Intern Med 40: 197–242. 7747647

[11] Sutherland LR, Church DL, Gill MJ, Kelly JK, Hwang WS, et al (1990) Gastrointestinal function and structure in HIV-positive patients. Cmaj 143: 641–646. 2207920 PMC1452325

[12] Church DL, Sutherland LR, Gill MJ, Visser ND, Kelly JK (1992) Absence of an association between enteric parasites in the manifestations and pathogenesis of HIV enteropathy in gay men. The GI/HIV Study Group. Scand J Infect Dis 24: 567–575. 1361241 10.3109/00365549209054642

[13] May GR, Gill MJ, Church DL, Sutherland LR (1993) Gastrointestinal symptoms in ambulatory HIV-infected patients. Dig Dis Sci 38: 1388–1394. 8102092 10.1007/BF01308593

[14] Carlson S, Yokoo H, Craig RM (1994) Small intestinal HIV-associated enteropathy: evidence for panintestinal enterocyte dysfunction. J Lab Clin Med 124: 652–659. 7964123

[15] Byakwaga H, Boesecke C, Emery S (2009) Inflammation and gut permeability. J HIV Ther 14: 57–60. 20218240

[16] Greenson JK, Belitsos PC, Yardley JH, Bartlett JG (1991) AIDS enteropathy: occult enteric infections and duodenal mucosal alterations in chronic diarrhea. Ann Intern Med 114: 366–372. 1992878 10.7326/0003-4819-114-5-366

[17] Ullrich R, Riecken EO, Zeitz M (1991) Human immunodeficiency virus-induced enteropathy. Immunol Res 10: 456–464. 1683358 10.1007/BF02919742

[18] Ullrich R, Zeitz M, Heise W, L'Age M, Hoffken G, et al (1989) Small intestinal structure and function in patients infected with human immunodeficiency virus (HIV): evidence for HIV-induced enteropathy. Ann Intern Med 111: 15–21. 2500046 10.7326/0003-4819-111-1-15

[19] Zeitz M, Ullrich R, Schneider T, Kewenig S, Hohloch K, et al (1998) HIV/SIV enteropathy. Ann N Y Acad Sci 859: 139–148. 9928377 10.1111/j.1749-6632.1998.tb11118.x

[20] Guadalupe M, Reay E, Sankaran S, Prindiville T, Flamm J, et al (2003) Severe CD4+ T-cell depletion in gut lymphoid tissue during primary human immunodeficiency virus type 1 infection and substantial delay in restoration following highly active antiretroviral therapy. J Virol 77: 11708–11717. 14557656 10.1128/JVI.77.21.11708-11717.2003 PMC229357

[21] Brenchley JM, Price DA, Schacker TW, Asher TE, Silvestri G, et al (2006) Microbial translocation is a cause of systemic immune activation in chronic HIV infection. Nat Med 12: 1365–1371. 17115046 10.1038/nm1511

[22] Chase A, Zhou Y, Siliciano RF (2006) HIV-1-induced depletion of CD4+ T cells in the gut: mechanism and therapeutic implications. Trends Pharmacol Sci 27: 4–7. 16337692 10.1016/j.tips.2005.11.005

[23] Douek D (2007) HIV disease progression: immune activation, microbes, and a leaky gut. Top HIV Med 15: 114–117. 17720995

[24] Sharkey KA, Sutherland LR, Davison JS, Zwiers H, Gill MJ, et al (1992) Peptides in the gastrointestinal tract in human immunodeficiency virus infection. The GI/HIV Study Group of the University of Calgary. Gastroenterology 103: 18–28. 1535325 10.1016/0016-5085(92)91090-q

[25] Spiller R (2007) Recent advances in understanding the role of serotonin in gastrointestinal motility in functional bowel disorders: alterations in 5-HT signalling and metabolism in human disease. Neurogastroenterol Motil 19 Suppl 2: 25–31. 17620085 10.1111/j.1365-2982.2007.00965.x

[26] Lomax AE, Linden DR, Mawe GM, Sharkey KA (2006) Effects of gastrointestinal inflammation on enteroendocrine cells and enteric neural reflex circuits. Auton Neurosci 126–127: 250–257. 10.1016/j.autneu.2006.02.015 16616704

[27] Van Op den Bosch J, Adriaensen D, Van Nassauw L, Timmermans JP (2009) The role(s) of somatostatin, structurally related peptides and somatostatin receptors in the gastrointestinal tract: a review. Regul Pept 156: 1–8. 19362110 10.1016/j.regpep.2009.04.003

[28] Gershon MD (2013) 5-Hydroxytryptamine (serotonin) in the gastrointestinal tract. Curr Opin Endocrinol Diabetes Obes 20: 14–21. 23222853 10.1097/MED.0b013e32835bc703 PMC3708472

[29] Coates MD, Mahoney CR, Linden DR, Sampson JE, Chen J, et al (2004) Molecular defects in mucosal serotonin content and decreased serotonin reuptake transporter in ulcerative colitis and irritable bowel syndrome. Gastroenterology 126: 1657–1664. 15188158 10.1053/j.gastro.2004.03.013

[30] Gill MJ, Sutherland LR, Church D (1992) Group TUoCGHs (1992) Gastrointestinal tissue cultures for HIV in HIV-infected/AIDS patients. AIDS 6: 553–556. 1388876 10.1097/00002030-199206000-00005

[31] al-Mulla W, Church D, Gill MJ (1997) Phenotypic variations and switches in HIV isolated from the blood and the gastrointestinal tissues of patients with HIV-1 infection. HIV/GI Research Study Group. J Med Virol 52: 31–34. 9131455

[32] van Marle G, Gill MJ, Kolodka D, McManus L, Grant T, et al (2007) Compartmentalization of the gut viral reservoir in HIV-1 infected patients. Retrovirology 4: 87. 18053211 10.1186/1742-4690-4-87 PMC2217557

[33] van Marle G, Church DL, Nunweiler KD, Cannon K, Wainberg MA, et al (2010) Higher levels of Zidovudine resistant HIV in the colon compared to blood and other gastrointestinal compartments in HIV infection. Retrovirology 7: 74. 20836880 10.1186/1742-4690-7-74 PMC2949729

[34] May GR, Sutherland LR, Meddings JB (1993) Is small intestinal permeability really increased in relatives of patients with Crohn's disease? Gastroenterology 104: 1627–1632. 8500719 10.1016/0016-5085(93)90638-s

[35] Hollinger FB, Bremer JW, Myers LE, Gold JW, McQuay L (1992) Standardization of sensitive human immunodeficiency virus coculture procedures and establishment of a multicenter quality assurance program for the AIDS Clinical Trials Group. The NIH/NIAID/DAIDS/ACTG Virology Laboratories. J Clin Microbiol 30: 1787–1794. 1629336 10.1128/jcm.30.7.1787-1794.1992 PMC265382

[36] Hanley JA, Negassa A, Edwardes MD, Forrester JE (2003) Statistical analysis of correlated data using generalized estimating equations: an orientation. Am J Epidemiol 157: 364–375. 12578807 10.1093/aje/kwf215

[37] Kotler DP (1998) Human immunodeficiency virus-related wasting: malabsorption syndromes. Semin Oncol 25: 70–75. 9625387

[38] Tersmette M, Gruters RA, de Wolf F, de Goede RE, Lange JM, et al (1989) Evidence for a role of virulent human immunodeficiency virus (HIV) variants in the pathogenesis of acquired immunodeficiency syndrome: studies on sequential HIV isolates. J Virol 63: 2118–2125. 2564898 10.1128/jvi.63.5.2118-2125.1989 PMC250628

[39] Miedema F, Meyaard L, Koot M, Klein MR, Roos MT, et al (1994) Changing virus-host interactions in the course of HIV-1 infection. Immunol Rev 140: 35–72. 7821927 10.1111/j.1600-065x.1994.tb00864.x

[40] Connor RI, Sheridan KE, Ceradini D, Choe S, Landau NR (1997) Change in coreceptor use coreceptor use correlates with disease progression in HIV-1–infected individuals. J Exp Med 185: 621–628. 9034141 10.1084/jem.185.4.621 PMC2196142

[41] Connor RI, Mohri H, Cao Y, Ho DD (1993) Increased viral burden and cytopathicity correlate temporally with CD4+ T-lymphocyte decline and clinical progression in human immunodeficiency virus type 1-infected individuals. J Virol 67: 1772–1777. 8095306 10.1128/jvi.67.4.1772-1777.1993 PMC240220

[42] Gershon MD, Liu MT (2007) Serotonin and neuroprotection in functional bowel disorders. Neurogastroenterol Motil 19 Suppl 2: 19–24. 17620084 10.1111/j.1365-2982.2007.00962.x PMC2832324

[43] Goyal RK, Hirano I (1996) The enteric nervous system. N Engl J Med 334: 1106–1115. 8598871 10.1056/NEJM199604253341707

[44] Mellors JW, Rinaldo CR Jr, Gupta P, White RM, Todd JA, et al (1996) Prognosis in HIV-1 infection predicted by the quantity of virus in plasma. Science 272: 1167–1170. 8638160 10.1126/science.272.5265.1167

[45] Chun TW, Carruth L, Finzi D, Shen X, DiGiuseppe JA, et al (1997) Quantification of latent tissue reservoirs and total body viral load in HIV-1 infection. Nature 387: 183–188. 9144289 10.1038/387183a0

[46] Haase AT (1999) Population biology of HIV-1 infection: viral and CD4+ T cell demographics and dynamics in lymphatic tissues. Annu Rev Immunol 17: 625–656. 10358770 10.1146/annurev.immunol.17.1.625

[47] Yukl SA, Gianella S, Sinclair E, Epling L, Li Q, et al (2010) Differences in HIV burden and immune activation within the gut of HIV-positive patients receiving suppressive antiretroviral therapy. J Infect Dis 202: 1553–1561. 20939732 10.1086/656722 PMC2997806

[48] Chun TW, Nickle DC, Justement JS, Meyers JH, Roby G, et al (2008) Persistence of HIV in gut-associated lymphoid tissue despite long-term antiretroviral therapy. J Infect Dis 197: 714–720. 18260759 10.1086/527324

[49] Deiman B, van Aarle P, Sillekens P (2002) Characteristics and applications of nucleic acid sequence-based amplification (NASBA). Mol Biotechnol 20: 163–179. 11876473 10.1385/MB:20:2:163

[50] Griffith BP, Rigsby MO, Garner RB, Gordon MM, Chacko TM (1997) Comparison of the Amplicor HIV-1 monitor test and the nucleic acid sequence-based amplification assay for quantitation of human immunodeficiency virus RNA in plasma, serum, and plasma subjected to freeze-thaw cycles. J Clin Microbiol 35: 3288–3291. 9399536 10.1128/jcm.35.12.3288-3291.1997 PMC230164

[51] Adachi A, Koenig S, Gendelman HE, Daugherty D, Gattoni-Celli S, et al (1987) Productive, persistent infection of human colorectal cell lines with human immunodeficiency virus. J Virol 61: 209–213. 3640832 10.1128/jvi.61.1.209-213.1987 PMC255241

[52] Schuitemaker H, Koot M, Kootstra NA, Dercksen MW, de Goede RE, et al (1992) Biological phenotype of human immunodeficiency virus type 1 clones at different stages of infection: progression of disease is associated with a shift from monocytotropic to T-cell-tropic virus population. J Virol 66: 1354–1360. 1738194 10.1128/jvi.66.3.1354-1360.1992 PMC240857

[53] Spijkerman I, de Wolf F, Langendam M, Schuitemaker H, Coutinho R (1998) Emergence of syncytium-inducing human immunodeficiency virus type 1 variants coincides with a transient increase in viral RNA level and is an independent predictor for progression to AIDS. J Infect Dis 178: 397–403. 9697719 10.1086/515627

[54] Koot M, van Leeuwen R, de Goede RE, Keet IP, Danner S, et al (1999) Conversion rate towards a syncytium-inducing (SI) phenotype during different stages of human immunodeficiency virus type 1 infection and prognostic value of SI phenotype for survival after AIDS diagnosis. J Infect Dis 179: 254–258. 9841850 10.1086/314539

[55] Troyer RM, Collins KR, Abraha A, Fraundorf E, Moore DM, et al (2005) Changes in human immunodeficiency virus type 1 fitness and genetic diversity during disease progression. J Virol 79: 9006–9018. 15994794 10.1128/JVI.79.14.9006-9018.2005 PMC1168764

[56] van Marle G, Power C (2005) Human immunodeficiency virus type 1 genetic diversity in the nervous system: evolutionary epiphenomenon or disease determinant? J Neurovirol 11: 107–128. 16036790 10.1080/13550280590922838

[57] Power C, McArthur JC, Nath A, Wehrly K, Mayne M, et al (1998) Neuronal death induced by brain-derived human immunodeficiency virus type 1 envelope genes differs between demented and nondemented AIDS patients. J Virol 72: 9045–9053. 9765449 10.1128/jvi.72.11.9045-9053.1998 PMC110321

[58] Wang H, Steeds J, Motomura Y, Deng Y, Verma-Gandhu M, et al (2007) CD4+ T cell-mediated immunological control of enterochromaffin cell hyperplasia and 5-hydroxytryptamine production in enteric infection. Gut 56: 949–957. 17303597 10.1136/gut.2006.103226 PMC1994360

[59] Motomura Y, Ghia JE, Wang H, Akiho H, El-Sharkawy RT, et al (2008) Enterochromaffin cell and 5-hydroxytryptamine responses to the same infectious agent differ in Th1 and Th2 dominant environments. Gut 57: 475–481. 18198200 10.1136/gut.2007.129296

[60] Yang GB, Lackner AA (2004) Proximity between 5-HT secreting enteroendocrine cells and lymphocytes in the gut mucosa of rhesus macaques (Macaca mulatta) is suggestive of a role for enterochromaffin cell 5-HT in mucosal immunity. J Neuroimmunol 146: 46–49. 14698846 10.1016/j.jneuroim.2003.10.044

[61] Costiniuk CT, Angel JB (2012) Human immunodeficiency virus and the gastrointestinal immune system: does highly active antiretroviral therapy restore gut immunity? Mucosal Immunol 5: 596–604. 22929559 10.1038/mi.2012.82

